# A Sporadic Juvenile Gastric Polyp: An Endoscopic Rarity

**DOI:** 10.1002/ccr3.71960

**Published:** 2026-03-01

**Authors:** Nisar Amin, Abdullahi Sulaiman, Ebubekir Daglilar, Harleen K. Chela

**Affiliations:** ^1^ Department of Internal Medicine Charleston Area Medical Center Charleston West Virginia USA; ^2^ Department of Pathology Charleston Area Medical Center Charleston West Virginia USA; ^3^ Department of Gastroenterology & Hepatology West Virginia University‐Charleston Division Charleston West Virginia USA

**Keywords:** endoscopy, gastroenterology/hepatology, general medicine, medical education, pathology

## Abstract

There are several types of gastric polyps and juvenile gastric polyps are scarce. They can be asymptomatic or can contribute to iron deficiency anemia. Can be sporadic or part of syndromic diagnosis. It's crucial to differentiate from other gastric polyps, as juvenile gastric polyps are rare with clinical implications.

## 1 Case

**FIGURE 1:**
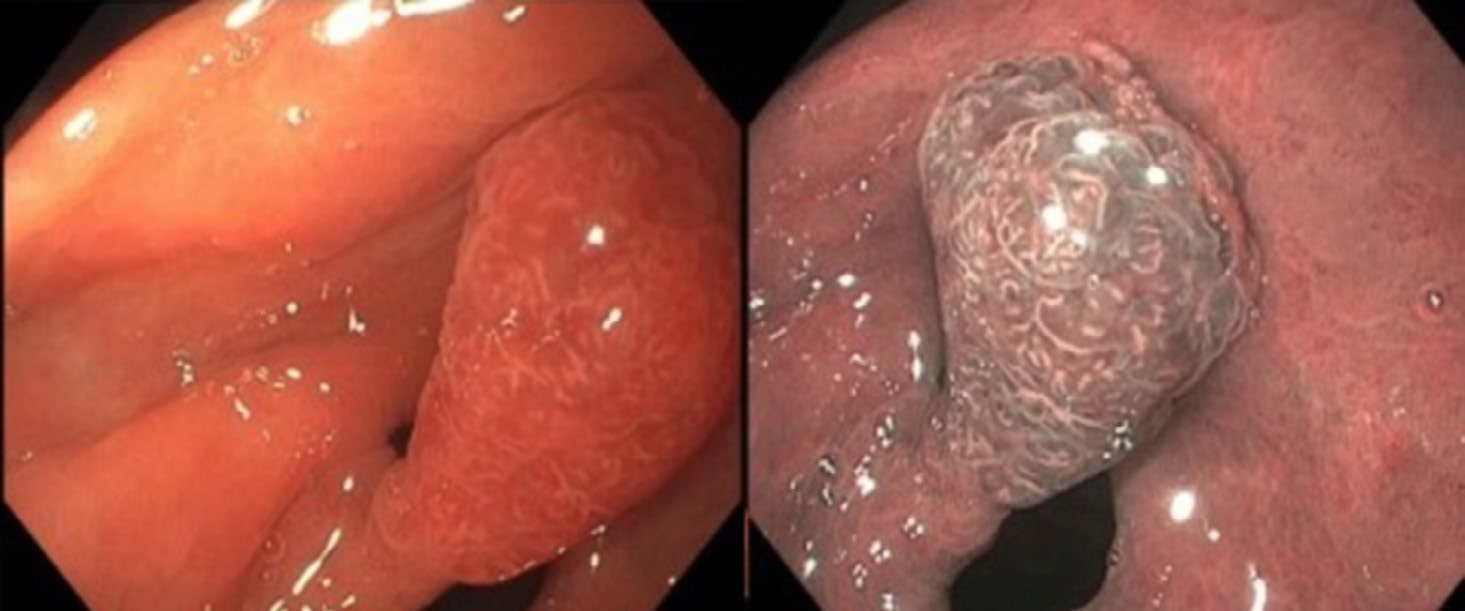
Endoscopy images showing the large pedunculated polyp in the gastric antrum under white light (on left) and narrow band imaging (on right).

**FIGURE 2:**
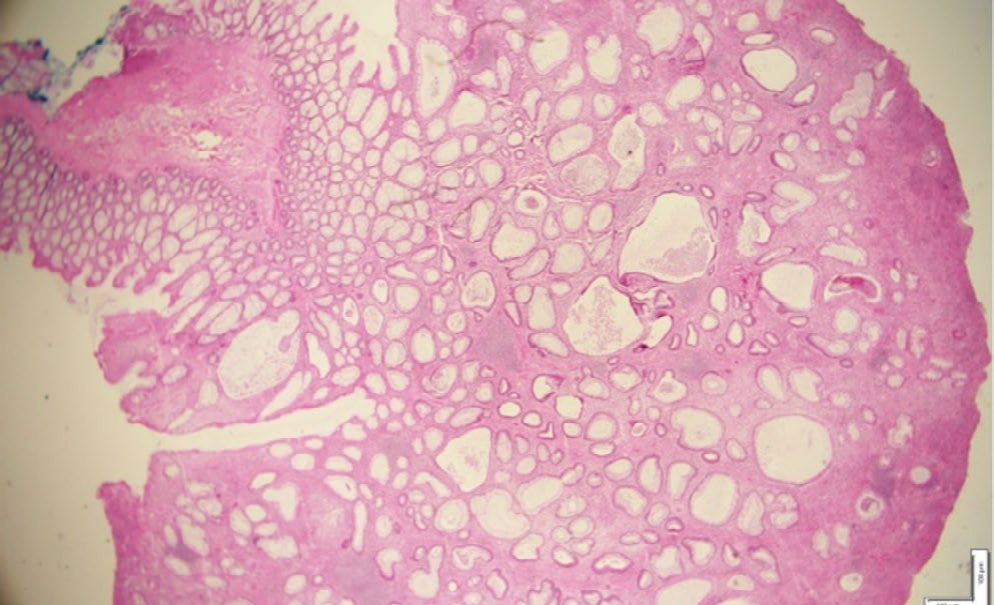
Histopathology images showing dilated glands lined by foveolar epithelial lining, with variable degree of inflammation.

**FIGURE 3:**
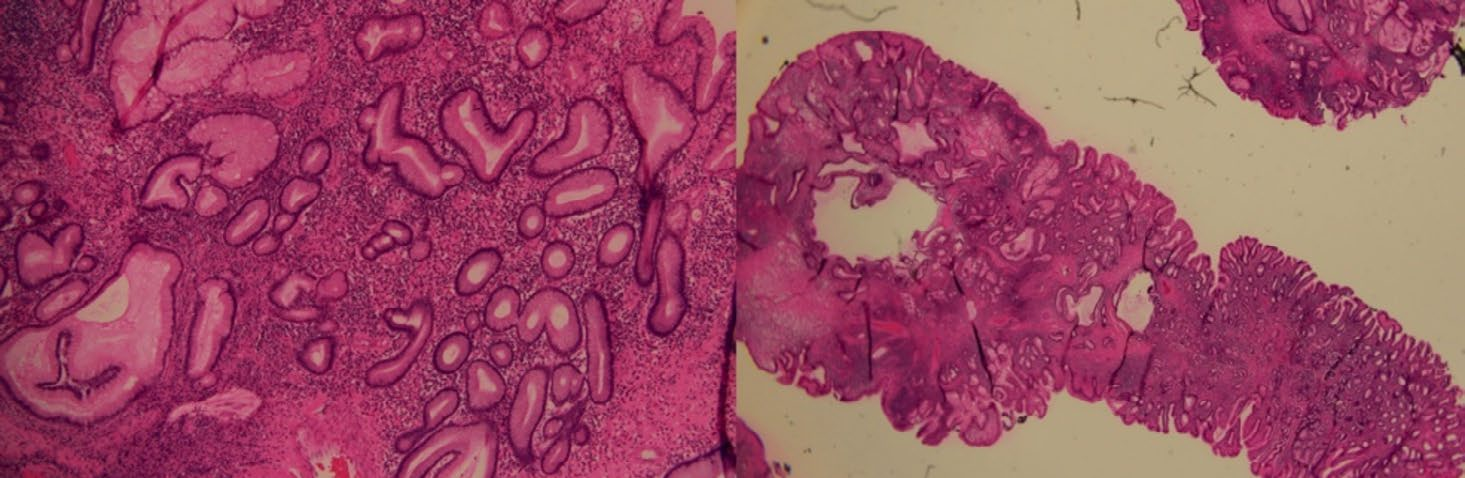
High‐powered view of polypoid lesion with multiple cystic gland lined by flattened epithelial cells with associated surface ulceration and variable lymphoplasmacytic infiltrate.

58‐year‐old male with no past medical history presented to hospital for abdominal pain, acute onset hematochezia. Family history was unremarkable for gastrointestinal malignancy or polyposis syndromes. Labs revealed mild normocytic anemia with hemoglobin of 10.8 g/dL. CT abdomen pelvis showed note of diverticulosis in sigmoid colon. Upper endoscopy and colonoscopy were performed, revealing diverticulosis in sigmoid colon, which was determined to be the likely cause of the hematochezia which had spontaneously resolved. On upper endoscopy, a large about 1.5 cm pedunculated gastric polyp in antrum (Figure 1) was seen, as well as mild gastritis in body and antrum. Polyp was removed completely and biopsies obtained separately to evaluate for *Helicobacter pylori* . Random gastric biopsies showed chronically active *Helicobacter pylori* gastritis, foci of intestinal metaplasia. Histopathology of polyp was consistent with juvenile polyp, showing dilated glands lined by foveolar epithelial lining, with variable degree of inflammation (Figure 2). High‐powered view of polypoid lesion with multiple cystic glands lined by flattened epithelial cells with associated surface ulceration and variable lymphoplasmacytic infiltrate (Figure 3). There was no evidence of dysplastic or neoplastic features in histopathology.

What type of polyp is this?

1. Hyperplastic polyp
2. Adenomatous polyp
3. Juvenile polyp
4. Fundic gland polyp

## 2 Discussion

Given histopathology findings, the polyp is a juvenile polyp. These are hamartomatous types of polyps and are often characterized by cystically dilated mucin‐filled glands that are lined by foveolar epithelium and inflamed edematous lamina propria [1]. Rarely seen as sporadic occurrence or can be a presentation of juvenile polyposis syndrome [1]. Sporadic juvenile polyps are rarely encountered in the stomach [1]. There are many different types of gastric polyps that are seen, and the most common are fundic gland type of polyps, which are essentially benign. Juvenile polyposis syndrome is the most common type of inherited gastrointestinal polyposis syndrome and is autosomal dominant [2]. Juvenile polyps are often discovered incidentally, and patients are asymptomatic; however, they can be associated with iron deficiency anemia and gastrointestinal bleeding [1, 2]. In our patient, diverticulosis was an incidental finding and the etiology of hematochezia. The juvenile polyp itself did not contribute to bleeding. These hamartomatous polyps can be located in the stomach, small intestine, colon, and rectum [2]. The syndrome is associated with germline mutations involving SMAD4 and BMPR1A [1]. Most juvenile polyps are benign but carry a risk for malignant transformation, and periodic endoscopic surveillance may be needed long term [3]. With regards to our patient, we offered outpatient follow‐up in clinic for genetic testing and further evaluation. However, he was unfortunately lost to follow‐up.

Classification systems are available for categorizing different types of gastric polyps. A recently developed classification published in 2024 describes gastric polyps as either: the good, the bad and the ugly [3]. These serve in aiding clinicians to recognize types of polyps. Some common gastric polyps that are encountered during endoscopy are fundic gland polyps that are benign in nature and associated with the use of proton pump inhibitors [3]. They are rarely associated with any malignant potential [3]. Hyperplastic polyps, however, are linked to the potential for dysplasia and neoplasia [3]. It is important to recognize types of polyps based on endoscopic features, though key to diagnosis is histopathology. Given the rarity of juvenile gastric polyps, it's crucial to be able to differentiate these from other types of gastric polyps. It is important to identify whether they are sporadic or part of a syndromic diagnosis and to consider genetic counseling accordingly.

## Author Contributions

**Nisar Amin:** conceptualization, investigation, visualization, writing – original draft, writing – review and editing. **Abdullahi Sulaiman:** visualization, writing – review and editing. **Ebubekir Daglilar:** investigation, writing – original draft, writing – review and editing. **Harleen K. Chela:** conceptualization, investigation, methodology, supervision, validation, visualization, writing – original draft, writing – review and editing.

## Funding

The authors have nothing to report.

## Consent

Witten and informed consent was obtained from the patient.

## Conflicts of Interest

The authors declare no conflicts of interest.

## Data Availability

The date is openly available to the public and the data for this manuscript are all included in the manuscript and the supporting documents itself. All the data used to create the manuscript is included and openly available.

## References

[1] A. Kang and A. K. Esnakula , “Juvenile Polyp,” PathologyOutlines.com website, accessed July 8, 2025, https://www.pathologyoutlines.com/topic/stomachjuvenile.html.

[2] J. Larsen Haidle , S. P. MacFarland , and J. R. Howe , “Juvenile Polyposis Syndrome,” [Updated 2022 Feb 3] in GeneReviews [Internet], ed. M. P. Adam , J. Feldman , G. M. Mirzaa , et al. (University of Washington, 2003), https://www.ncbi.nlm.nih.gov/books/NBK1469/. 20301642

[3] D. Costa , D. Ramai , and A. Tringali , “Novel Classification of Gastric Polyps: The Good, the Bad and the Ugly,” World Journal of Gastroenterology 30, no. 31 (2024): 3640–3653, 10.3748/wjg.v30.i31.3640. 39192997 PMC11346164

